# Tactical and statistical analysis of spiking efficiency by type, zone and set phase in women's volleyball

**DOI:** 10.3389/fspor.2025.1630870

**Published:** 2025-08-22

**Authors:** Kiattisak Sitti, K. Ravivuth Rangubhet

**Affiliations:** ^1^ Faculty of Education, Thailand National Sport University Sukhothai Campus, Sukhothai, Thailand; ^2^ Department of Exercise and Sport Science, Faculty of Science, University of Phayao, Phayao, Thailand

**Keywords:** tactical analysis, performance indicators, spike type, attack zone, set phase, volleyball tactics

## Abstract

**Background:**

Spiking is a decisive offensive action in elite women's volleyball, with variations in spike type, court zone, and timing influencing match outcomes. Understanding tactical and temporal dimensions of spiking can offer insights into offensive efficiency and performance consistency.

**Methods:**

A total of 2,599 spike attempts were analyzed from 29 matches (108 sets) in the 2024 Women's Volleyball Nations League. Each spike was categorized by type (straight, deep, diagonal, block-out, tip), attack zone (side vs. central), and set phase (early: Sets 1–2; middle: Set 3; endgame: Sets 4–5). Key performance indicators included kill rate, error rate, and efficiency index (EI). Comparative statistics were used to assess performance differences across match outcomes, spike types, zones, and phases.

**Results:**

Block-out spikes achieved the highest overall efficiency (EI = 0.79). Diagonal spikes showed significantly greater efficiency in winning sets (EI = 0.346) than in losing sets (EI = 0.147, *p* = 0.0198). Tip shots had the lowest efficiency (EI = 0.22) but the highest continuation rate (61%). Side zone attacks exhibited higher kill rates (25.2%) and efficiency (EI = 0.227) than central zone attacks (14.1% kill rate, EI = 0.106). Performance peaked during the middle set phase (EI = 0.318) but declined in the endgame phase (EI = 0.195; error rate = 0.151).

**Conclusions:**

Spike type selection, court positioning, and set phase timing significantly affect offensive performance. These insights can help coaches and analysts refine tactical planning, player rotation, and energy distribution strategies in elite women's volleyball.

## Introduction

Volleyball is a dynamic team sport that demands technical precision, tactical coordination, and physical endurance. Among its core skills, spiking is recognized as the most direct and decisive action in determining rally outcomes and final match results (1, 2). In recent years, performance analysis in volleyball has expanded beyond quantitative counts of actions, incorporating efficiency metrics, spatial zoning, and temporal dynamics to assess individual and team effectiveness (3, 4).

**Figure 1:**
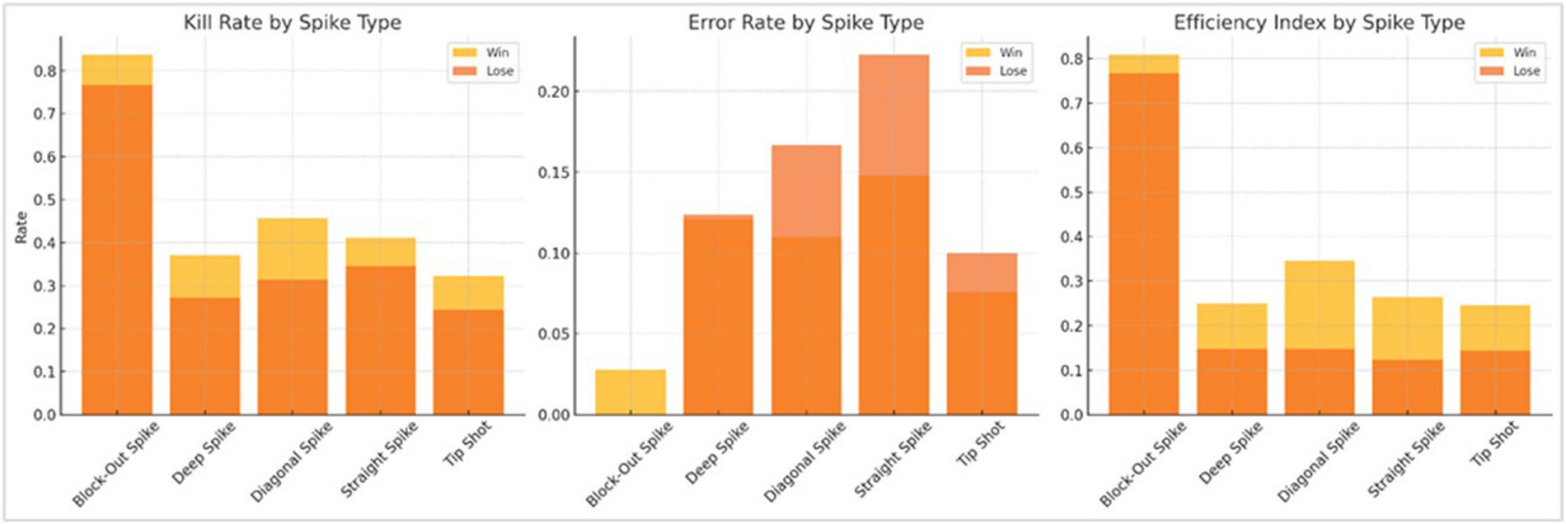
Comparison of spiking metrics—kill rate, error rate, and efficiency Index (EI)—across five spike types, separated by match outcome (win vs. lose). Diagonal spikes showed the largest performance gap between winning and losing sets, with significantly higher EI (*p* = 0.0198) in wins. Block-out spikes maintained the highest EI overall (>0.76) with minimal error, while tip shots showed improved kill rate in winning contexts.

Various methodological approaches have been employed to evaluate tactical performance in elite volleyball. Traditional analysis often relied on box-score statistics and skill frequency counts, which, while objective, failed to capture the contextual and interactive nature of in-game decisions [A]. More advanced systems integrate notational analysis, game complex evaluations, and movement patterns, offering a broader tactical perspective [B]. For instance, the “game complex” framework—dividing play into offensive and defensive phases—has been used to analyze player transitions and team efficiency within each sequence (5). Other approaches involve the modeling of team systems, such as 5-1 or 6-2 formations, and their impact on offensive coordination and attack timing (6). Despite their strengths, these models also present challenges. Complex coding frameworks may suffer from low inter-rater reliability, while oversimplified models may obscure nuanced interactions between spike type, zone selection, and opponent strategy (7). Therefore, tactical performance evaluation must balance data granularity with practical interpretability for coaches and analysts.

In Southeast Asian volleyball research, Rangubhet has played a pivotal role in developing analytical frameworks for tactical performance evaluation. His studies have demonstrated that spiking effectiveness varies by spike type, execution zone, and match context (8). The integration of spatial zone indicators—particularly side and central attack zones—has proven valuable for identifying performance trends and designing training protocols for elite athletes (9). Zone-based models are now used to assess offensive efficiency and tactical adaptation in competitive matches (10).

International literature also emphasizes the situational nature of spiking success. For instance, López-Martínez et al. found that variability in spike execution and block response significantly affects performance outcomes (11). Similarly, Silva et al. reported that spiking efficiency is influenced by team rotation, reception quality, and block structure (12). These findings underscore the need to analyze spiking as a multidimensional action embedded within match dynamics.

The efficiency index (EI)—calculated as (Kills−Errors)/Total Spike Attempts—has become a standard metric for evaluating offensive performance (13). Recent applications by Rangubhet and colleagues have extended EI across spike types (e.g., straight, diagonal, block-out, tip) and tactical zones, revealing key differences in spiking performance between winning and losing teams (14). These insights form the basis for data-driven coaching decisions, particularly in elite women's volleyball, where small tactical shifts can yield meaningful competitive advantages.

Given this background, the present study aims to investigate spiking efficiency in elite women's volleyball by analyzing spike type, court zone, and set phase. Through tactical and statistical approaches, this research seeks to identify performance patterns that contribute to successful outcomes. The findings are expected to inform offensive strategy optimization and support training interventions in high-performance volleyball contexts.

## Materials and methods

### Design

Our analysis reveals employed a quantitative observational design to analyze spiking performance in elite women's volleyball through tactical and statistical lenses. The investigation focused on three primary variables: spike type, court attack zone, and match phase. A retrospective approach was utilized, drawing on official match data without experimental manipulation, to preserve the authenticity of performance conditions. This methodology aligns with established practices in performance analysis research (1, 2).

### Participants/sample

#### Match selection

The dataset comprised 29 official matches from the 2024 Women's Volleyball Nations League, specifically selecting the champion and runner-up teams to capture spiking behaviors under elite, high-stakes conditions. This purposive sampling strategy focused on high-performance tactical execution, consistent with prior research emphasizing critical competitive moments (3).

#### Spiking instances

From the 108 sets analyzed, a total of 2,599 spiking attempts were identified. Each spiking event was reviewed from match footage and coded manually by trained analysts according to:

• Spike type: straight, deep, diagonal, block-out, and tip shot
• Outcome: kill (point scored), error (direct fault), or neutral (continuation)
• Court zone: side attack zones or central attack zone
• Set phase: early (sets 1–2), middle (set 3), late (sets 4–5)
• Match result: win or loss

This classification approach followed frameworks proposed by Rangubhet et al. (15) and other leading performance analysis models.

#### Data collection

##### Court zone classification

The net area of the volleyball court was divided into three 3-meter longitudinal segments. For tactical analysis, these were consolidated into two zones:

• Side Attack Zones: the left and right flanks near the sidelines, typically used for diagonal, deep, and tip shots, including plays like “Y”, “curve”, “back Y”, and “cross-line”.
• Central Attack Zone: the middle segment, often used for fast-paced attacks such as “quick”, “overlap”, and “slide”.

This zoning system was grounded in both theoretical positioning models and real-match tendencies observed in elite-level play (16).

##### Set phase classification

The match timeline was segmented into three phases:

• Early phase: Sets 1 and 2
• Middle phase: Set 3
• Late phase: Sets 4 and 5

This classification was designed to evaluate performance variation over time, accounting for factors such as fatigue, momentum, or tactical adjustments during match progression (14).

#### Performance indicators

##### Independent variables

• Spike Type (categorical: 5 types)
• Attack Zone (categorical: side, central)
• Set Phase (ordinal: early, middle, late)
• Match Outcome (binary: win, lose)

##### Dependent variables/performance metrics

• Kill Rate = Kills/Total Spike Attempts
• Error Rate = Errors/Total Spike Attempts
• Efficiency Index (EI) = (Kills−Errors)/Total Spike Attempts
• Continuation Rate = Number of Neutral Outcomes/Total Spike Attempts

This metric represents the proportion of spikes that did not result in immediate point gain (kill) or error but continued the rally.

• Average Net Efficiency (per phase) = Mean of [(Kills−Errors)/Spike Attempts] calculated separately for each set and then averaged across sets within a phase

These indicators are widely accepted in performance analysis research and allow meaningful comparison across tactical conditions (10, 11).

### Statistical analysis

#### Data preparation

Spiking data were manually entered and verified in Microsoft Excel before being imported into IBM SPSS Statistics (Version 28) and Python (Pandas and Matplotlib) for statistical analysis and visualization. Coding accuracy was reviewed through inter-observer checks, ensuring reliability of the dataset. Inter-rater reliability was assessed on 15% of the dataset using percentage agreement, achieving over 92% consistency among three trained analysts.

#### Descriptive analysis

Means, standard deviations, and percentages were calculated to summarize spiking performance by spike type, zone, and match phase.

#### Inferential statistics

Data normality was assessed using the Shapiro–Wilk test. The results showed that all key performance indicators (Kill Rate, Error Rate, Efficiency Index) for both winning and losing sets followed a normal distribution (*p* > .05), justifying the use of parametric tests where applicable. Table X presents the detailed normality results.

To assess performance differences:

• Depending on distribution and sample size, either independent-sample *t*-tests or Mann–Whitney *U*-tests were applied to compare kill rate, error rate, and efficiency index across winning and losing outcomes.
• The significance level was set at *p* < .05 for all analyses.

#### Data visualization

To support interpretation and tactical application, the study also generated:

• Radar charts (spike type usage by zone)
• Bar charts (scoring vs. error rates)
• Zone-based summary tables and performance efficiency grids

These visual tools were used to present performance trends in an accessible format for applied use by coaches and performance analysts.

### Ethical considerations

This study was approved by the Human Research Ethics Committee of the University of Phayao (Protocol No. HREC-UP-HSST 1.1/044/67). All procedures adhered to the principles of the Declaration of Helsinki. As the study involved retrospective analysis of publicly available match footage, no direct involvement or consent from human subjects was required.

## Results

Table 1 distribution of spike performance outcomes classified as successful and unsuccessful spikes, based on data from 29 matches and 108 sets during the 2024 Women's Volleyball Nations League. Successful spikes include kill spikes and scoring outcomes resulting from ineffective opponent responses. Unsuccessful spikes include execution errors and spikes blocked resulting in immediate point loss (block errors), which are reported separately for clarity, as recommended by the reviewer.

**Table 1:** Spike performance report categorized by outcome, based on 29 matches and 108 sets in the 2024 women's volleyball nations league.

Spike outcome	Frequency (*n*)	Mean per match	SD
Successful spikes	1,534	7.30	6.58
– Kill spike	571	19.03	5.74
– Opponent unable to return (first contact failure)	329	10.97	4.41
– Opponent receives but fails to continue play	119	3.97	2.93
– Ball deflected outside attacker's right side	84	2.80	1.85
– Ball deflected outside attacker's back	62	2.07	1.36
– Ball deflected outside defender's left side	132	4.40	2.36
– Block contact but ball lands out of bounds	237	7.90	3.62
Unsuccessful spikes	421	3.51	3.94
– Spike error (missed contact)	12	0.40	0.77
– Out of bounds	170	5.67	2.56
– Into the net	30	1.00	0.91
– Blocked resulting in point loss (Block error)	209	6.97	4.70

Table 2 presents descriptive and inferential analyses of key spiking performance metrics—kill rate, error rate, and net efficiency—aggregated across all sets and stratified by match outcome.

**Table 2:** Overall comparison of spiking metrics by match outcome.

Metric	Overall mean	Overall SD	Win mean	Win SD	Lose mean	Lose SD	*p*-value
Kill rate	0.393	0.080	0.420	0.071	0.344	0.073	0.0003*
Error rate	0.125	0.054	0.115	0.046	0.142	0.063	0.0807
Efficiency index	0.268	0.107	0.305	0.082	0.201	0.115	0.0007*

* Mean and standard deviation (SD) are reported for each metric across all sets and match outcomes. Data normality was assessed using the Shapiro–Wilk test, and *p*-values were calculated using Mann–Whitney *U*-tests based on the results of the normality assessment. Statistically significant differences (*p* < 0.05) are marked with an asterisk.

The overall kill rate was 0.393 (SD = 0.080). A significant difference was found between winning and losing sets, with winning sets showing a higher kill rate (M = 0.420, SD = 0.071) than losing sets (M = 0.344, SD = 0.073), *p* = 0.0003. This suggests that successful teams were more proficient in converting attacks into direct points, reinforcing the importance of terminal offensive execution (1, 2).

The net efficiency index further emphasized this disparity, averaging 0.305 (SD = 0.082) in winning sets compared to 0.201 (SD = 0.115) in losing sets (*p* = 0.0007). This substantial difference highlights the role of both scoring and error minimization in achieving competitive advantage, consistent with frameworks proposed by Marcelino et al. (13) and expanded by Rangubhet (15).

Although the error rate was higher in losing sets (M = 0.142) than in winning sets (M = 0.115), the difference did not reach statistical significance (*p* = 0.0807). However, the interaction between kill rate and error rate—captured by the net efficiency index—emphasizes the importance of using composite indicators to assess offensive quality, rather than relying on isolated metrics (17).

Overall, the findings underscore that spiking efficiency is a critical predictor of match success and should be prioritized in coaching strategies, performance diagnostics, and player evaluations.

Table 3 presents the performance characteristics of five spike types used during the tournament, including usage frequency, Kill rate, error rate, continuation rate, and efficiency index (EI). Clear differences were observed in both usage patterns and offensive effectiveness.

**Table 3:** Performance summary of spike types.

Spike type	Used (n)	Kill rate	Error rate	Continuation rate	Efficiency index (EI)
Deep spike	817	0.34	0.12	0.48	0.22
Straight spike	622	0.39	0.17	0.38	0.23
Diagonal spike	471	0.41	0.13	0.44	0.28
Tip shot	478	0.30	0.08	0.61	0.22
Block-out spike	211	0.82	0.02	0.13	0.79

Efficiency Index (EI) = (Success−Errors)/Total Attempts. Continuation Rate refers to the proportion of spike attempts that resulted in neutral outcomes, representing rally continuation.

Deep spikes were the most frequently used (*n* = 817), with moderate success (0.34) and high continuation rate (0.48), indicating their role in sustaining rallies and adjusting to defensive setups. Straight spikes showed slightly higher efficiency (EI = 0.23) but also the highest error rate (0.17), suggesting greater execution risk.

Diagonal spikes yielded the highest Kill rate among commonly used types (0.41) and a solid efficiency index (0.28), reflecting a favorable balance between scoring potential and risk. These findings support their tactical reliability and confirm their value in scoring during key match phases, especially in winning sets (14, 16).

Tip shots, while the least aggressive in terms of scoring (Kill rate = 0.30; EI = 0.22), exhibited the highest continuation rate (0.61). This underscores their role as tactical disruptors—deployed to extend rallies, break blocking rhythm, or exploit gaps under pressure—consistent with previous studies on soft spiking tactics (12).

Block-out spikes stood out as the most efficient type, with an outstanding Kill rate of 0.82, minimal errors (0.02), and a remarkably high EI of 0.79. Although used less frequently (*n* = 211), their consistent scoring output highlights their strategic utility in bypassing blocks and minimizing risk (17).

These findings underline the importance of spike-type-specific tactical planning in elite women's volleyball. Coaches are encouraged to:

• Prioritize block-out and diagonal spikes in scoring-focused drills and match planning.
• Incorporate tip shots as situational tools, particularly in late sets or against structured blocks.
• Tailor training to balance technical precision with situational awareness, optimizing both offensive output and rally control.

Continuation Rate refers to the proportion of spike attempts that resulted in neutral outcomes (i.e., not immediate kills or errors), reflecting rally continuation. Average Net Efficiency is calculated as the mean of EI values across all sets within each match phase.

Table 4 summarizes spiking performance across different set phases—early (Sets 1–2), middle (Set 3), and endgame (Sets 4–5)—highlighting both output and variability indicators.

**Table 4:** Spiking performance across Set phases with additional tactical indicators.

Set phase	Avg total spikes	Avg kills	Avg errors	Avg kill rate	Avg error rate	Avg net efficiency	Net efficiency SD	Success/Error ratio	% high efficiency sets
Early	65.83	25.63	7.63	0.397	0.116	0.281	0.090	4.10	50.0%
Middle	66.67	28.33	7.47	0.433	0.114	0.318	0.084	4.41	66.7%
Endgame	35.19	12.13	5.00	0.346	0.151	0.195	0.122	3.23	18.8%

“High Efficiency Sets” are defined as sets with a net efficiency above the overall mean. EI = (Kills−Errors)/Total Spike Attempts. Average Net Efficiency was calculated per set and then averaged across all sets within each match phase to reflect phase-specific trends.

The middle phase demonstrated the highest offensive performance, with a peak kill rate of 0.433, net efficiency of 0.318, and success/error ratio of 4.41. Two-thirds of sets (66.7%) in this phase were classified as high-efficiency, suggesting this period represents tactical and physical optimality. Likely contributing factors include improved rhythm, setter-attacker synchronization, and stabilized tactics following initial adjustments (17).

In contrast, the endgame phase showed noticeable decline: kill rate dropped to 0.346 and error rate increased to 0.151—the highest among all phases. Consequently, this phase recorded the lowest net efficiency (0.195) and lowest percentage of high-efficiency sets (18.8%), suggesting fatigue-induced or pressure-related performance degradation (3, 18). Such declines are supported by previous analyses that show skill execution in late sets especially under pressure tends to deteriorate due to cumulative fatigue and tactical adaptation by opponents (18). Additionally, net efficiency variability (SD = 0.122) was highest in this phase, indicating greater inconsistency.

The early phase produced intermediate values, with a kill rate of 0.397 and EI of 0.281. This phase may reflect the transitional stage before players and teams fully adapt to match flow and opponent strategies.

The inclusion of success/error ratio and % high-efficiency sets provides further granularity beyond standard metrics. These indicators emphasize not only raw scoring output but also attack balance and performance consistency, which are crucial for high-level tactical planning.

### Practical implications

• Coaches should leverage the middle phase as a tactical advantage zone, emphasizing offensive schemes and targeted spike types.
• To counter endgame decline, conditioning programs and substitution strategies should focus on maintaining energy and technical accuracy under fatigue.
• Monitoring phase-based performance may support in-match decision-making and post-match evaluations.

Table 5 compares spiking performance by spike type across winning and losing sets. Among the five spike types, diagonal spikes showed statistically significant differences in both kill rate (*p* = 0.013) and efficiency index (*p* = 0.0198), favoring winning sets. This confirms diagonal spikes as a decisive tactical option, combining angular trajectory and moderate risk to maximize scoring under pressure (14, 16).

**Table 5:** Spike type performance by match outcome with statistical comparison.

Spike type	Attempts (Lose)	Attempts (Win)	Kill rate (Lose)	Kill rate (Win)	Error rate (Lose)	Error rate (Win)	Efficiency (Lose)	Efficiency (Win)	*p*-value (Kill Rate)	*p*-value (Error Rate)	*p*-value (EI)
Block-out spike	61	150	0.767	0.838	0.000	0.028	0.767	0.810	0.720	0.146	0.632
Deep spike	253	564	0.272	0.370	0.124	0.121	0.148	0.249	0.256	0.722	0.210
Diagonal spike	146	325	0.314	0.456	0.167	0.110	0.147	0.346	0.013	0.103	0.019
Straight spike	156	466	0.345	0.412	0.223	0.148	0.122	0.264	0.144	0.239	0.207
Tip shot	154	324	0.244	0.323	0.100	0.076	0.144	0.247	0.045	0.177	0.078

*p*-values calculated using Mann–Whitney *U*-tests. Bolded values indicate statistical significance (*p* < 0.05). EI = (Kills – Errors)/Total Attempts.

**Table 6:** Shapiro–Wilk test for normality of performance variables.

Variable	Shapiro–Wilk statistic	*p*-value	Normality
Kill rate (Win)	0.9682	0.7155	Normal
Kill rate (Lose)	0.9472	0.3272	Normal
Error rate (Win)	0.9660	0.6681	Normal
Error rate (Lose)	0.9362	0.2027	Normal
Efficiency index (Win)	0.9458	0.3077	Normal
Efficiency index (Lose)	0.9426	0.2853	Normal

This table presents the results of normality testing using the Shapiro–Wilk test for each performance metric across match outcomes.

**Table 7:** Spike zone performance summary with tactical indicators.

Zone	Total spikes	Kill rate (%)	Efficiency index	Straight spike (%)	Deep spike (%)	Diagonal spike (%)	Block-out (%)	Tip shot (%)
Side attack zone	2,040	25.2%	0.227	27.6%	37.6%	10.4%	5.6%	18.8%
Central attack zone	559	14.1%	0.106	21.0%	33.8%	20.5%	7.5%	17.3%

Kill Rate = Kills/Total Spikes; Efficiency Index = (Kills−Errors)/Total Spikes. Percentages of spike types represent tactical distribution by zone.

Tip shots also demonstrated a significant difference in kill rate between winning and losing conditions (*p* = 0.0456), although differences in error rate and efficiency index were not statistically significant. Their role appears to be context-dependent, functioning effectively as tactical disruptors during successful play sequences (12).

Block-out spikes maintained the highest efficiency overall (EI = 0.810 in wins; 0.767 in losses), but did not show statistically significant differences. Their consistently low error rate—especially zero in losing sets—reinforces their reliability as a “safe scoring option” regardless of match context (17).

While straight and deep spikes were used frequently, their differences between winning and losing sets did not reach statistical significance. Nevertheless, the trend toward higher values in wins suggests their tactical value remains relevant, especially when integrated with speed and timing.

### Coaching and application insights

• Diagonal spikes should be emphasized in training for decisive scoring, particularly during high-leverage points.
• Tip shots can serve as tactical weapons to disrupt rhythm and exploit defensive overcommitment, but should be deployed opportunistically.
• Block-out spikes should remain a staple option for high-efficiency attacks under both pressure and recovery conditions.
• Coaches should evaluate not only spike frequency but contextual effectiveness to inform game planning and substitution strategies.
• For kill rate, winning teams outperformed losing teams across all spike types. The largest difference was observed in diagonal spikes, where the kill rate rose from 0.314 in losing sets to 0.456 in winning sets. A similar pattern was seen in tip shots (0.244 vs. 0.323).
• Regarding error rate, all spike types showed lower error rates in winning sets. Notably, block-out spikes yielded error rates in both win (0.028) and loss (0.000) conditions, reaffirming their tactical reliability.
• For efficiency index, diagonal spikes again demonstrated the greatest gap between outcomes (0.147 in losses vs. 0.346 in wins), followed by straight and tip spikes, both showing a increase in winning sets. Block-out spikes consistently showed the highest EI (>0.76). Figure 1 illustrates the comparison of kill rate, error rate, and efficiency index across spike types for winning and losing sets.

This visualization confirms the tactical and statistical importance of diagonal spikes in match success, as they exhibit the most significant improvements in both scoring efficiency and kill rate in winning contexts (14, 16).

Block-out spikes, while maintaining the highest efficiency overall, did not show a significant difference between match outcomes—suggesting their consistent reliability. Their exceptionally low error rate supports their use as a “safe” finishing strategy in high-pressure situations.

Tip shots, often seen as defensive or deceptive plays, contributed more effectively in winning sets. The increase in kill rate and EI suggests that judicious use of tip shots may serve as a critical tactical disruptor, especially when opposing defenses overcommit to anticipated power attacks (12).

It underscores the need for tactical diversity and spike-type-specific preparation in elite volleyball, especially under match conditions that require both precision and adaptability.

Table 5 illustrates the performance and distribution of spike types across two tactical court zones: Side attack zones and Central attack zone. Clear contrasts in offensive output.

### Side attack zones

Side Attack Zone exhibited superior offensive output, with a higher kill rate (25.2%) and efficiency index (0.227) compared to Central Attack Zone. The distribution of straight (27.6%) and deep spikes (37.6%) was highest in this zone, suggesting a preference for linear, high-velocity attacks exploiting court width. This pattern is consistent with strategies employed by outside hitters seeking to bypass tight block formations with speed and placement (16).

### Central attack zone

Despite a higher frequency of total attempts (*n* = 559), Zone yielded a lower kill rate (14.1%) and efficiency index (0.106), potentially due to more predictable timing and centralized defensive positioning. However, this zone showed greater use of diagonal (20.5%) and block-out spikes (7.5%), indicating an adaptive response—favoring angled attacks and tool-based finishes against blocks. These tactical adjustments align with findings on mid-zone countering techniques in elite volleyball (17).

### Tip shot usage

Tip shots were deployed almost equally across both zones (∼18%), reflecting their situational role rather than zone-specific preference. Their usage supports tactical disruption and control rather than aggressive termination, particularly under defensive pressure (12).

### Coaching applications

• Side Attack Zone attackers should emphasize depth, power, and court-wide angles to maximize scoring.
• Central Attack Zone strategies may benefit from increased training on tempo variation, angle generation, and block manipulation.
• Zone-specific spike-type distribution can inform positional drills, match strategy, and role assignments for outside vs. middle hitters.

**Figure 2:**
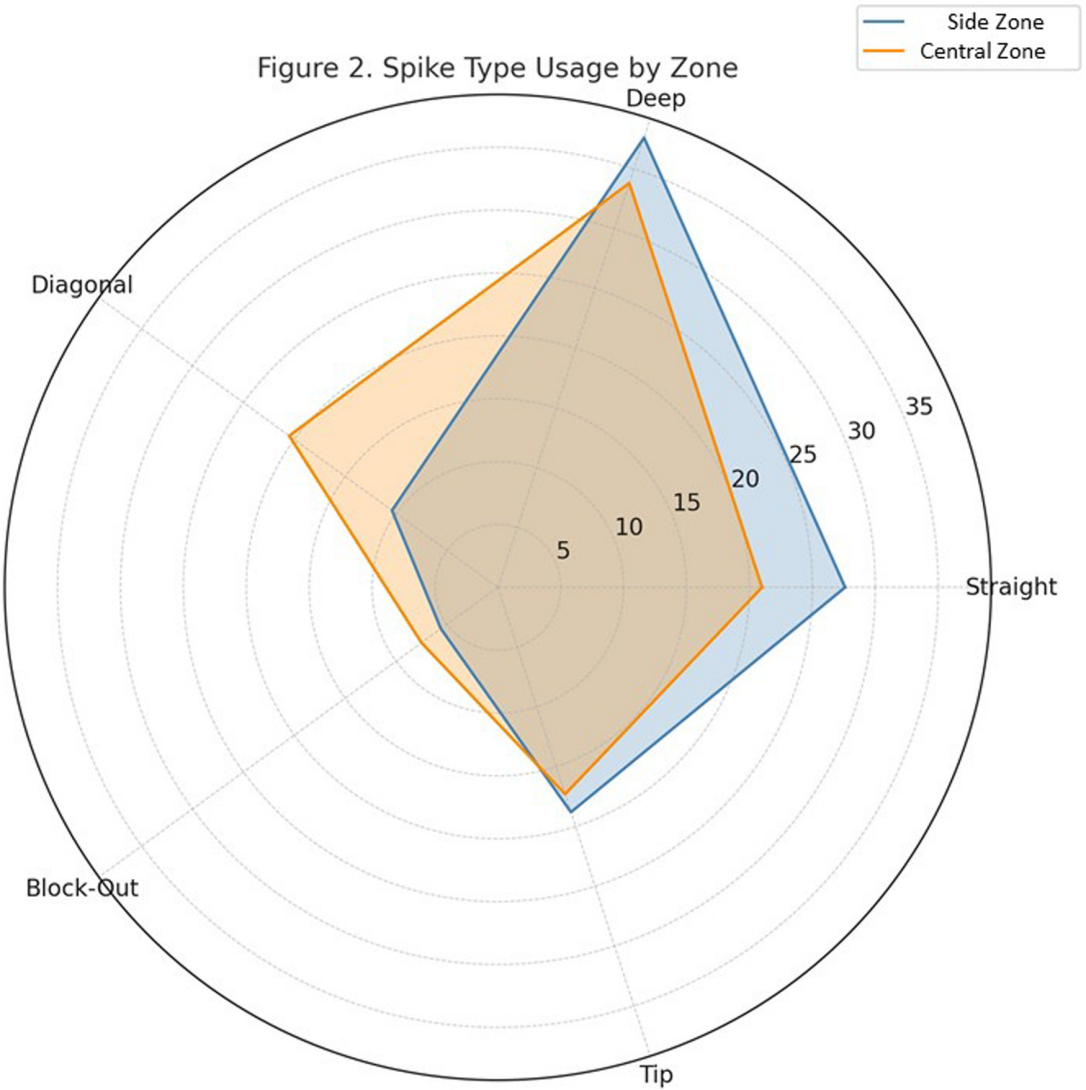
Radar chart showing the distribution of five spike types—straight, deep, diagonal, block-out, and tip—across two tactical court zones: Side attack zone and central attack zone. Side attack zone favored straight and deep spikes (25.2% kill rate, EI = 0.227), while central attack zone saw more diagonal and block-out usage, reflecting positional adaptation to block structures.

Figure 2 Illustrates the distribution of five spike types—straight, deep, diagonal, block-out, and tip shots—used in the two tactical zones. Notable differences in spike selection patterns can be observed between the two zones.

• Side Attack Zone showed higher usage rates of straight spikes (27.6%) and deep spikes (37.6%), indicating a reliance on power-based and line-trajectory attacks from the flanks.
• In contrast, Central Attack Zone displayed greater use of diagonal spikes (20.5%) and block-out spikes (7.5%), reflecting more angular and tactical spike options from central positions.
• The distribution of tip shots was relatively balanced across both zones (18.8% in Side Attack Zone vs. 17.3% in Central Attack Zone.

This radar chart highlights clear tactical tendencies linked to spike-type selection across court zones. These patterns are consistent with tactical zoning literature, which emphasizes the role of spike tempo and angular trajectory in optimizing offensive outcomes based on court position (17). This zone is typically accessed by outside hitters (13), who often exploit broader angles and use depth to bypass defensive blocks.

Conversely, the higher proportions of diagonal and block-out spikes in central attack zone suggest a more nuanced approach. Central attackers appear to rely more on angle variation and block manipulation, possibly due to quicker sets and tighter spacing. The elevated use of block-out spikes in this zone aligns with findings by Sitti and Rangubhet (14), who emphasized the importance of controlled deflections in tight net battles.

These insights support zone-specific training approaches where spike-type selection is aligned with positional tendencies and opponent defensive structures.

## Discussion

The present analysis explored spiking performance in elite women's volleyball by examining three critical tactical dimensions: spike type, attack zone, and set phase. The findings provide meaningful insights into how these variables interact to influence offensive efficiency, underlining their importance for match success and informed strategic planning.

A key finding was the superior effectiveness of block-out spikes, which demonstrated the highest efficiency index (EI = 0.79) and the lowest error rate (2%). Although their frequency of use was relatively low, their high reliability across both winning and losing sets suggests they serve as a low-risk, high-reward tactical tool. This supports the notion that controlled deflection strategies can enhance point-scoring opportunities in net confrontations (17). In contrast, diagonal spikes exhibited statistically significant differences between winning and losing sets (*p* = 0.0198), affirming their role as a context-sensitive offensive weapon. These results corroborate prior findings emphasizing the angular advantages and temporal flexibility of diagonal attacks when facing well-structured blocks (16).

Tip shots—often regarded as secondary options—emerged as a valuable component of the offensive arsenal, demonstrating higher continuation rates and greater impact in winning scenarios than previously assumed. This is consistent with evidence indicating increased tip utilization under pressure to disrupt defensive rhythm and prolong rallies (12). Such outcomes reinforce the importance of situational adaptability and tactical variation in elite-level play.

Spatial analysis revealed that attacks from side zones were associated with higher kill rates and efficiency indices compared to central zone attacks. This finding supports earlier work showing that wider court angles offer superior attacker-blocker positioning and increased scoring potential (5). The present study extends these insights by illustrating that straight and deep spikes were dominant from side zones, while diagonal and block-out spikes were more prevalent from central zones—reflecting adaptive offensive behaviors in response to geometric and opponent block constraints.

Temporally, performance varied across match phases. Efficiency peaked in the middle set (EI = 0.318), likely reflecting improved team coordination following initial tactical adjustments. A notable decline in efficiency and a rise in errors were observed in the endgame phase (EI = 0.195), suggesting the compounded effects of fatigue, psychological pressure, and opponent adaptation. These patterns echo previous findings linking late-game performance decrements to stress-related declines in motor control and decision-making accuracy (18). Accordingly, coaches may benefit from monitoring these dynamics to optimize substitutions and energy conservation.

A notable strength of this research lies in its multidimensional approach, integrating tactical, spatial, and temporal variables across a robust dataset from high-level matches. However, certain limitations should be acknowledged. The sample was limited to two top-performing teams in the 2024 Women's Volleyball Nations League, which may constrain the generalizability of findings. Additionally, contextual variables such as reception quality, attack tempo, and blocker positioning were not included, although they likely influence spiking outcomes. Future research should incorporate these variables and adopt integrated models that reflect both offensive and defensive interactions.

These findings imply that coaches and analysts may benefit from incorporating spike-type–specific training and phase-sensitive tactical strategies into their development programs. Training should emphasize the development of diagonal and block-out spikes in scoring scenarios, while integrating tip shot drills in pressure simulations. Normality testing results are presented in Table 6. Zone-specific spike tendencies can also inform offensive schemes and player assignments. Tracking performance across match phases may support timely tactical adjustments and fatigue management.

Ultimately, the findings contribute to the growing body of volleyball performance literature by demonstrating that spiking efficiency is not merely a function of technical execution, but a dynamic outcome shaped by the interaction of spike type, spatial location, and temporal context. These findings can guide more nuanced, data-informed coaching strategies in elite competition.

Furthermore, the results align with emerging perspectives on tactical sustainability in volleyball. Low-risk, tactically disruptive actions such as jump float serves have been shown to stabilize team performance over time under dynamic match conditions (19). Similarly, identifying and training high-efficiency spike types reflects a broader strategic commitment to risk-managed, repeatable offensive patterns. Moreover, the integration of equity and inclusivity in performance planning—as highlighted in the context of athletic development (20) —suggests that diversifying player roles and empowering secondary options, like tip shots, may enhance both team cohesion and individual development. These integrated perspectives support not only tactical optimization but also athlete sustainability and engagement in high-performance environments.

## Conclusions

A key insight from the data is offers a comprehensive tactical and statistical analysis of spiking performance in elite women's volleyball, focusing on spike types, attack zones, match phases, and outcome-based comparisons. The findings affirm that spiking efficiency—particularly the integration of kill rate and error minimization—is a critical determinant of match success. Winning teams consistently demonstrated higher net efficiency and kill rates, especially through the effective use of diagonal and block-out spikes.

Among the five spike types analyzed, diagonal spikes emerged as the most outcome-sensitive, exhibiting statistically significant improvements in both scoring and efficiency in winning sets. While block-out spikes achieved the highest overall efficiency, their consistent performance across both win and loss conditions highlighted their tactical reliability, though with limited discriminatory power. Tip shots, though less dominant in scoring, contributed strategically by maintaining rallies and exploiting defensive gaps under pressure.

Zone-based analysis revealed that side attack zones generated superior offensive output, with higher kill rates and efficiency indices compared to central attack zones. Radar chart findings further demonstrated that spike-type preferences varied by zone: straight and deep spikes predominated in Side Attack Zone, while diagonal and block-out spikes were more prevalent in Central Attack Zone (Table 7), suggesting nuanced, location-dependent tactical execution.

Performance also varied by match phase, with the middle phase (Set 3) showing the highest spiking efficiency, while the endgame phase was marked by increased error rates and reduced consistency—likely influenced by fatigue or tactical adaptation by the opposition.

Taken together, these results underscore the importance of spike-type-specific, zone-aware, and phase-sensitive tactical planning. Coaches and performance analysts should go beyond volume-based evaluations to incorporate contextual indicators that reflect the spatial and temporal complexities of match play. This study contributes actionable, data-driven insights that support the development of refined offensive strategies and enhanced performance diagnostics in high-level women's volleyball.

## Data Availability

The raw data supporting the conclusions of this article will be made available by the authors, without undue reservation.
